# Association Between rs3833912/rs16944 SNPs and Risk for Cerebral Palsy in Mexican Children

**DOI:** 10.1007/s12035-018-1178-6

**Published:** 2018-06-21

**Authors:** Sofia Torres-Merino, Hayde Nallely Moreno-Sandoval, Maria del Rocio Thompson-Bonilla, Josselyn Alejandra Orendain Leon, Eduardo Gomez-Conde, Bertha Alicia Leon-Chavez, Daniel Martinez-Fong, Juan Antonio Gonzalez-Barrios

**Affiliations:** 1 Neuropediatric Department, Comprehensive Rehabilitation Center “CRIT-Telethon”, 224 Via Gustavo Baz Prada, CP 54015 Tlanepantla, State of Mexico Mexico; 2 Genomic Medicine Laboratory, Regional Hospital “October 1st”, ISSSTE, 1669 National Polytechnic Institute Ave, CP 07760 Mexico City, Mexico; 3 Immunology Research Laboratory, Medicine Faculty, BUAP, 14 south and San Claudio Ave, 72570 Puebla, Puebla Mexico; 4 Academy of Biochemistry and Molecular Biology, Chemistry Faculty, BUAP, 14 south and San Claudio Ave, 72570 Puebla, Puebla Mexico; 5 Department of Physiology, Biophysics and Neurosciences, CINVESTAV, 2508 National Polytechnic Institute Ave, CP 06760 Mexico City, Mexico

**Keywords:** Cerebral palsy, NOS2A, IL-1β, Polymorphism, Microsatellite, Haplotype

## Abstract

Perinatal asphyxia in the neonatal brain triggers a robust inflammatory response in which nitric oxide (NO) generation plays a hazardous role. Increased levels of NO can be maintained by the activity of inducible NO synthase (NOS2A) on its own or activated by IL-1beta (IL-1β) gene transcription and positive back stimulation of the NOS2 (CCTTT)n microsatellite by IL-1β, thus potentiating brain injury after ischemic perinatal asphyxia. We investigated whether the risk for cerebral palsy (CP) increases when an expansion of the − 2.5 kb (CCTTT)n microsatellite in the NOS2A gene and a single nucleotide polymorphism (SNP) in -C511T of the IL- IL-1β gene promoter occur in patients after perinatal hypoxic-ischemic encephalopathy. Genomic DNA was purified from peripheral leukocytes of 48 patients with CP and of 57 healthy control children. IL-1β SNP genotypes were established using a real-time PCR technique and fluorogenic probes and were validated by restriction fragment length polymorphism (RFLP) analysis using the AvaI restriction enzyme. The length of the CCTTTn microsatellite in the NOS2 gene promoter was determined by automated sequencing. The 14 repeat-long allele of the CCTTTn NOS2A microsatellite was present in 27% of CP patients vs 12.3% of controls, showing an odds ratio (OR) = 2.6531 and 95% confidence interval (CI) = 0.9612–7.3232 (*P* < 0.0469). The -511 TT genotype frequency showed an OR = 2.6325 (95% CI = 1.1348–6.1066, *P* = 0.0189). Interestingly, the haplotype CCTTT_14_/TT showed an OR = 9.561 (95%, CI = 1.1321–80.753; *P* = 0.0164). The haplotype (CCTTT)_14_/TT, formed by the expansion of the − 2.5 kb (CCTTT)_n_ microsatellite in the NOS2A gene promoter and the -511 C➝ T SNP of the IL-1β gene promoter, might be a useful marker to identify patients who are at high risk for developing CP after hypoxic-ischemic encephalopathy.

## Introduction

Intrauterine, partum, and neonatal asphyxia results in hypoxic-ischemic encephalopathy (HIE), which is the leading cause of cerebral palsy (CP) in developing countries [1]. During cerebral hypoxia insult, the production of nitric oxide (NO) increases the levels of peroxynitrites [2] that, together with superoxides [3, 4], cause neuronal damage by inducing lipid peroxidation, chromatinorrhexis, and structural protein degradation [5]. Of the three NO synthase isoforms (NOS1, NOS2, and NOS3) [6–8], only NOS2 expression can be induced by cerebral hypoxia insult [8, 9] and is known to be responsible for the hazardous levels of NO in some animal models [10]. NOS2 is a family of three inducible genes (NOS2A, NOS2B, and NOS2C) that are localized in human chromosome 17 [11]. Only the promoter of NOS2A at position − 2.5 kb presents the expansion of pentanucleotide polypyrimidine microsatellite CCTTT (rs3833912) that has been associated with NO overproduction [12], which depends on the number of repeats of the motif. Specifically, the (CCTTT)_13–15_ alleles increase NO production by 3.5- and 4-fold in vitro [13]. Interestingly, the 13, 14, and 15 microsatellite repeats are activated by interleukin-1 beta (IL-1β) protein under hypoxic and inflammatory conditions, thus drastically increasing NO production [13, 14]. IL-1β is a proinflammatory cytokine that plays a crucial role in neuroinflammation of the HIE [15, 16] and can induce neurodegeneration after an ischemic injury [17]. Previous studies have determined that the specific haplotype T allele at − 511 and C allele at − 31 in the IL-1β gene promoter increase the production and release of IL-1β by two- to threefold under inflammatory conditions [18]. Thus, patients carrying the single nucleotide polymorphism (SNIP) at the − 511 position of the IL-1β gene promoter together with the expansion of the CCTTT microsatellite in the NOS2A promoter might be more susceptible to develop CP after HIE. Our main aim was to explore this possibility by comparing CP children with healthy control children.

## Materials and Methods

### Subjects

This case and control study included 105 children (40 females and 65 males) aged from 7 to 16 years with an antecedent of gestation older than 35 weeks. The case group included 48 patients with a history of perinatal asphyxia, low Apgar score (< 7 at 5 min), stage II or III Sarnat score in the neonatal period, and no records of chorioamnionitis or maternal infection. All patients were diagnosed with CP and were treated in the Neuropediatrics Department of the Comprehensive Rehabilitation Center “Telethon” (CRIT) in Tlalnepantla, Estado de Mexico. The control group included 57 children who were selected based on the following criteria: healthy condition with no neurological antecedents, electroencephalogram (EEG) in the normal range, and computerized axial tomography (CAT) without malformation or cerebral damage, conducted specifically during the genomic study. A group that was low on Apgar score and Sarnat II but did not develop CP was not included because in Mexico this kind of patient rarely continues with medical care and insufficient records of neurological sequelae were available. The protocol was approved by the Institutional Review Board of the Comprehensive Rehabilitation Center “Telethon” and of the “October 1st” Regional Hospital, ISSSTE. Informed written consent was obtained from all of the parents of CP patients and healthy children for blood sample extraction and result publication.

### Clinical and Neurological Evaluation

The diagnosis of CP was established by a neuropediatrician staff member of the pediatric neurology department of CRIT based on clinical criteria from the American Neurology Association and the clinical manifestations described elsewhere [19, 20]. The CAT scans and their evaluations were performed by the staff of the Diagnostic Tomography Department with Brilliance CT 40-channel equipment (Philips; Bothell, WA, USA) in all CP patients. Waking EEG recordings were carried out in all CP patients with the absence of sedative drugs using only eight channels of disk-scalp electrodes placed according to the 10–20 International System. The impedance values of skin-electrodes were measured and were ≤ 20 kW. The EEG was recorded by a Nicolet Viking Quest equipment (Viasys Healthcare Corporate; San Diego, CA, USA) under standard criteria.

### Genotyping

#### iNOS Microsatellite Sequencing (rs3833912)

Peripheral whole blood was obtained from a finger on the left hand with a Contact-Activated Lancet (Becton, Dickinson and Company, Ozorków, Poland) and collected in a microtainer tube containing ethylenediaminetetraacetic acid (EDTA) as an anticoagulant (Becton, Dickinson and Company, Franklin Lakes, NJ, USA). Genomic DNA was isolated from leukocytes with a DNA Illustra blood genomicPrep Mini Spin Kit (General Electric Healthcare, Buckinghamshire, UK). Polymerase chain reaction (PCR) amplification of the corresponding fragments from the NOS2A promoter region was performed with a 9700 thermal cycler (Applied Biosystems, Foster City, CA, USA). To amplify the genomic region surrounding the (CCTTT)_n_ polymorphic microsatellite, the following primers were used: 5′-ACC CCT GGA AGC CTA CAA CTG CAT-3′ and 5′-GCC ATC GCA CCC TAG CCT GTC TCA-3′. The reaction was carried out in a final volume of 25 μL, which included PCR buffer 1X, 100 μM dNTPs, 200 μM of each primer, 500 μM MgSO_4_, 0.2 U of Pfx DNA polymerase, and 20 ng of genomic DNA. The following cycling conditions were used: initial denaturation at 95 °C for 5 min, followed by 35 cycles of 95 °C for 30 s, 65 °C for 30 s, and 72 °C for 1 min with a final extension at 72 °C for 10 min. Negative controls were included in each PCR reaction to confirm the absence of contamination. Samples were electrophoresed in 2% agarose gels at 100 V and 400 mA for 2 h to separate heterozygous alleles, and then, DNA was recovered by gel purification using a QIAquick Gel Extraction Kit (Qiagen Inc., Valencia, CA, USA). Purified PCR products were used to sequence PCR products using GenomeLab Methods Development Kit Dye Terminator Cycle Sequencing (Beckman Coulter; CA, USA). Sequencing reaction products were purified using an Agencourt CleanSEQ kit (Beckman Coulter, CA, USA) and subjected to direct sequencing electrophoresis in an automated Beckman CEQ8000 Genetic Analyzer DNA sequencer (Beckman Coulter, CA, USA). The resulting chromatograms were viewed and aligned using Chromas 2.3 (Technelysium Pty Ltd., Ewantin, Australia). The GenBank accession number for the reference genomic sequence of NOS2A is AF440785.

### IL-1 β Polymorphism

The single nucleotide polymorphism (SNP) -C511T (rs16944) genotype was evaluated by PCR amplification of the relevant fragment followed by identification using restriction fragment length polymorphism (PCR-RFLP). In summary, PCR for the rs16944 polymorphism was carried out using forward (5-TGG CAT TGA TCT GGT TCA TC-3) and reverse (5-GTT TAG GAA TCT TCC CAC TT-3) primers. Amplification was performed in a 25-μL tube containing 2.5 μL of 10X PCR buffer (100 mM Tris-HCI, 15 mM MgCl_2_, and 500 mM KCl, pH 8.3), 200 nM each dNTP (Invitrogen, Carlsbad, CA, USA), 1 M each primer (Invitrogen, Carlsbad, CA, USA), 1 U Taq DNA polymerase (Invitrogen, Carlsbad, CA, USA), and 10 ng of genomic DNA. The PCR conditions were 94 °C for 5 min followed by 35 cycles of 94 °C for 30 s, 60 °C for 30 s, 72 °C for 30 s, and finally 72 °C for 10 min. The -511 PCR products were digested with an AvaI restriction enzyme (New England Biolabs, Ipswich, MA, USA) at 37 °C overnight. Genotypes were designated as follows: T/T, two bands of 100 and 173 bp; C/T, three bands of 100, 173, and 273 bp; and C/C, a single band of 273 bp [21]. Validation of the rs16944 polymorphisms was carried out by genotyping using a Fluorogenic 5-nuclease assay (TaqMan allelic discrimination test). Primers and SNP-specific dual fluorogenic probes were labeled with 6-carboxyfluorescein (FAM), VIC as a reporter, and TAMRA as a quencher (Applied Biosystems; Warrington Cheshire, UK). Real-time PCR was carried with the forward primer 5′-CCC TTT CCT TTA ACT TGA TTG TGA AAT-3′ and the reverse primer 5′-TCT CTA CCT TGG GTG CTG TTC T-3′, while the C probe used was 5′-(VIC)CTG CCT CGG GAG CT(NFQ)-3′ and the T probe was 5′-(FAM)CTG CCT CAG GAG CT(NFQ)-3′ [18]. The real-time PCR cycling conditions were as follows: 50 °C for 2 min and 95 °C for 10 min, followed by 40 cycles of 95 °C for 15 s (denaturing) and 62 °C for 30 s (annealing) and 72 °C for 30 s (extension) using a 7900HT Fast Real-Time PCR System (Applied Biosystems, Foster City, CA, USA). The different alleles were discriminated according to the fluorescence intensity of Fam and Vic.

### Statistical Analysis

The difference in frequency distributions of genotypes between the CP patients and healthy controls was analyzed with PLINK version v1.07-1 and R v0.5.3 open-source software (GNU license). The latter software was used to test the possible association of the rs3833912 and rs16944 polymorphisms with CP. Relative risk (RR), odds ratios (ORs), and the 95% confidence interval (CI) were calculated by logistic regression analysis. *P* < 0.05 was considered statistically significant. Differences in the allele and genotype frequencies between the CP patients and the healthy controls and Hardy-Weinberg equilibrium were tested with a *χ*^2^ test. All data analyses were performed with the Linux operating system (Debian Squeeze).

## Results

### Selection of Study Subjects

The CP group was composed of 48 children who met the following criteria: (1) term product with more than 36 gestation weeks, (2) record of perinatal hypoxia/ischemia in neonatal medical records, (3) no genetic diseases, and (4) no brain malformations. The healthy control group was composed of 57 children who met the following criteria: (1) matching age and gender with respect to the CP group and (2) no record of neurological diseases in a clinical pediatric neurology evaluation.

### Age and Gender

The CP group (*n* = 48) consisted of 71% males and 29% females, whereas the healthy control group (*n* = 57) included 46% males and 54% females. The mean values and standard deviations (*s*) of ages were 11 (*s* = 2 years) years for patients with CP and 10 (*s* = 3) years for the control group. The statistical analysis did not show significant differences when the ages of the CP patients were compared with those of the control group (*P* = 0.6318; CI 95%, Student’s *t* test).

### Apgar Score

**Fig. 1:**
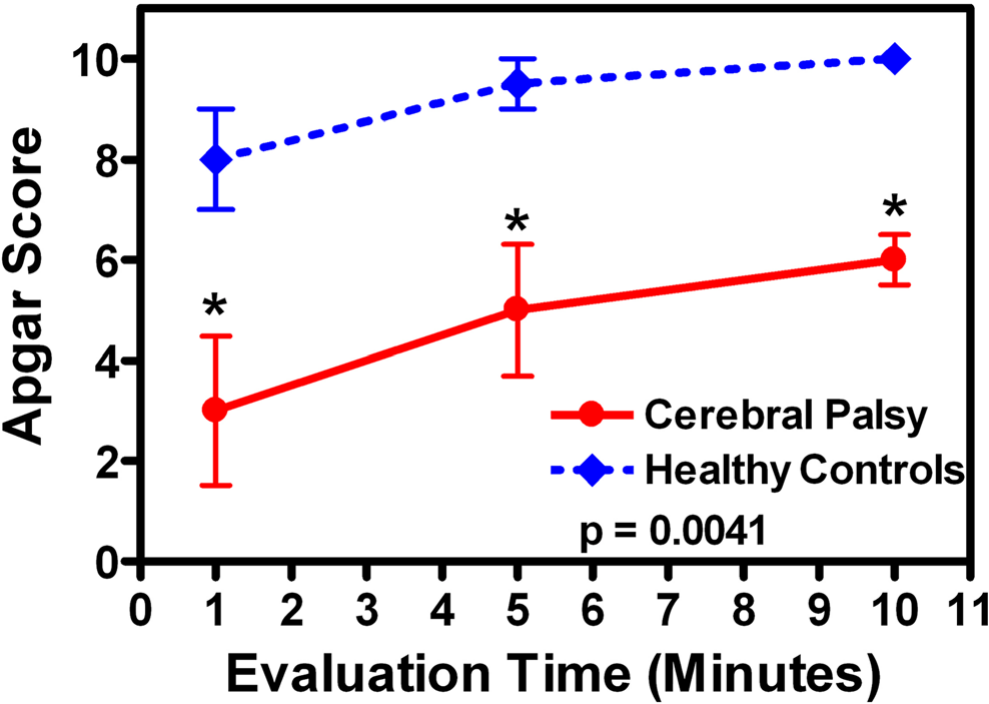
Time course of the Apgar score evaluation. All data were collected from medical records. *P < 0.5 compared with healthy controls; *χ*^2^ test

The diagnosis of perinatal hypoxia-ischemia in Mexico is mainly based on clinical variables, despite the increasing number of first-level clinical centers with the infrastructure to analyze blood pH in newborn patients. The main clinical diagnostic criteria of perinatal hypoxia-ischemia are the presence of neurological symptoms during the perinatal period (convulsions, coma, muscle hypotonia, and multiple-organ dysfunction syndrome) accompanied by a low Apgar score (5 or less) 10 min after delivery. This study was focused on patients who developed CP and had a history of an Apgar score < 7 at 5 min after delivery. In addition, 73% (*n* = 35) of our CP patients had a neonatal record of cord blood gases. The main results were pH 6.81 ± 0.13, pCO_2_ 106 ± 3.06 mmHg, and HCO3–13 ± 1.3 mmol/L. No data of cord blood gases for participants of the healthy control group were found in the neonatal records. The progressive Apgar scores at 1, 5, and 10 min after delivery of CP patients were significantly lower than those of the healthy controls at the three evaluations (Fig. 1). The Apgar scores of CP patients were 38% (minute 1), 53% (minute 5), and 60% (minute 10) with respect to the Apgar scores of healthy controls (Fig. 1).

### Clinical Findings

The severity of hypoxia-ischemia and individual genetic traits are the main factors that determine the different clinical subtypes of CP [19]. The analysis of neonatal records (Table 1) showed that 73% (*n* = 35) of CP patients were Sarnat stage II, which remained stable during the 8 ± 5-day stay in the neonatal intensive care unit (NICU), whereas 27% (*n* = 13) were classified as Sarnat stage III, with a longer stay (18 ± 7 days) in the NICU. The pediatric neurological evaluation at the CRIT-Telethon at enrollment showed that quadriparesis (81%) was the most frequent finding, followed by hemiparesis (8.8%), diparesis (5.3%), and other types of palsy (5.2%). The spastic subtype was the most common manifestation present in quadriparesis (54%), hemiparesis (100%), and diparesis (66%) in Mexican children with CP (Table 2).

**Table 1:** Summary of neonatal clinical data

	CP (*n* = 48)	HC (*n* = 57)	*P* value
Parameter			
Gender (M/F)	34/14	26/31	–
Gestational age (weeks)	39 ± 1.2	39.2 ± 1.6	0.001
Delivery			
Standard vaginal delivery	89.6%	93.1%	–
Emergency Cesarean section	10.4%	0%	–
Pre-programmed Cesarean section	0%	6.9%	
Delivery complications			
Fetal decelerations	54.2%	2.1%	–
Dystocic delivery	27.1%	2.1%	–
Nuchal cord	10.4%	4.2	–
Delivered at street	2.1%	ND	–
Cord prolapse	6.3%	ND	–
Apgar score			
1 min	3.0 ± 1.5	8.0 ± 0.5	< 0.000
5 min	5.0 ± 1.3	9.5 ± 0.5	< 0.000
10 min	6.0 ± 0.5	10	< 0.000
Sarnat staging			
Sarnat 2	73%	ND	–
Sarnat 3	27%	ND	–
Seizures	100%	ND	–
Cord blood gas			
pH	6.81 ± 0.13^a^	ND	–
pCO_2_	106 ± 3.06 mmHg^a^	ND	–
HCO3-	13 ± 1.3 mmol/L^a^	ND	–
Electroencephalographic findings			
Multiple seizures	25.0%	ND	–
Low amplitude, seizures, rare spikes	18.7%	ND	–
Low voltage rare spikes	16.7%	ND	–
Bilateral epileptiform spikes	16.7%	ND	–
Sharp-waves	10.4%	ND	–
Rare spikes; mild suppression	8.3%	ND	–
Severe suppression	4.2%	ND	–

*ND* no data available

^a^Patient with cord blood gas (*n* = 35)

**Table 2:** Motor disabilities in Mexican children with cerebral palsy secondary to perinatal hypoxia-ischemia

Diagnosis	Global	Type	Individual
Quadriparesis	80.7% (*n* = 39)	Spastic	54.3% (*n* = 21)
		Mixed flaccid	28.2% (*n* = 11)
		Athetosis	6.5% (*n* = 3)
		Dystonic	2.5% (*n* = 1)
		Flaccid	2.5% (*n* = 1)
		Dyskinetic	2.5% (*n* = 1)
		Spastic/dyskinetic	2.5% (*n* = 1)
Hemiparesis	8.8% (*n* = 4)	Right spastic	50% (*n* = 2)
		Double spastic	25% (*n* = 1)
		Left spastic	25% (*n* = 1)
Diparesis	5.3% (*n* = 3)	Spastic	66% (*n* = 2)
		Mixed	34% (*n* = 1)
Others	5.2% (*n* = 2)		

The data were obtained from the medical records of patients with cerebral palsy (CP) who received medical health care in the CRIT-Tlalnepantla

### Computerized Axial Tomography

**Fig. 2:**
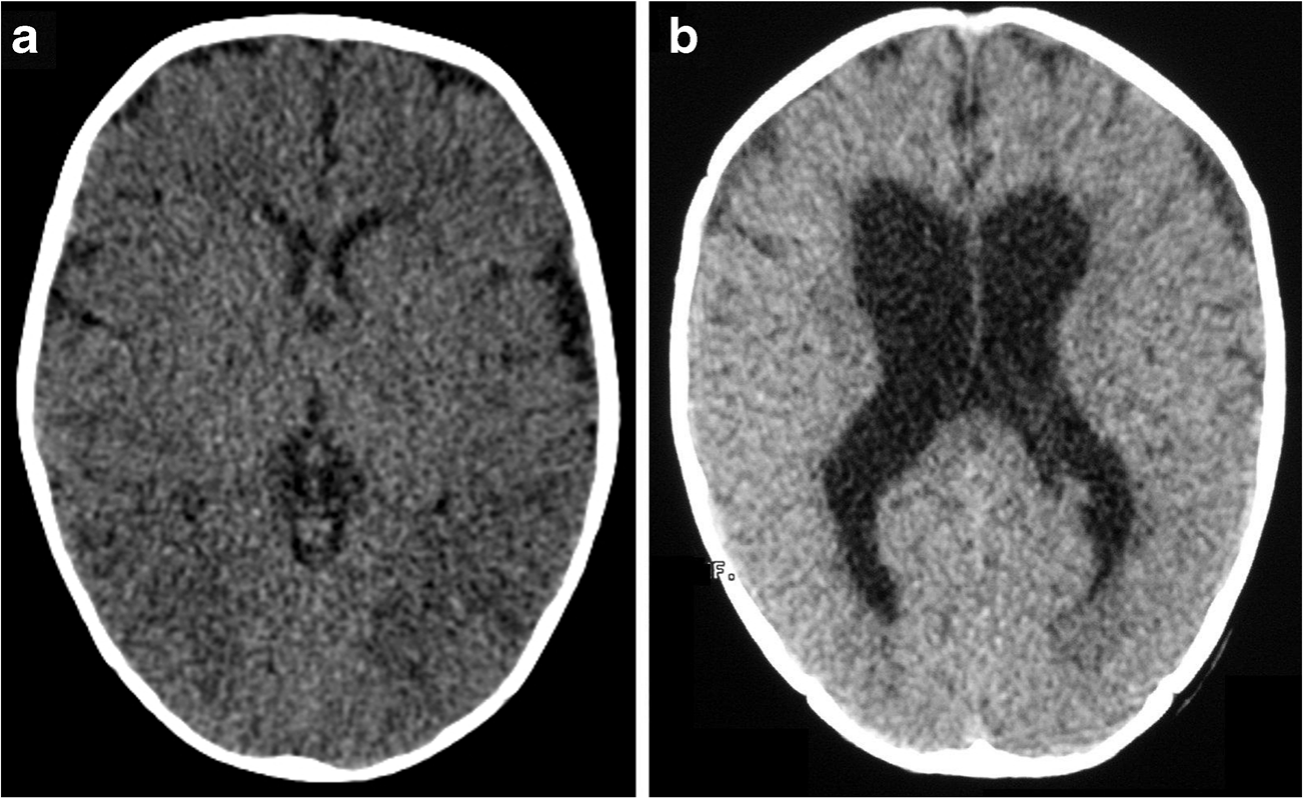
Representative brain images of computerized axial tomography (CAT) scans. **a** Patient with cerebral palsy. **b** Healthy control child

Neonatal CAT scans were not available for the CP patients; however, the current CAT analysis of all CP patients showed severe cortical atrophy; increased volume of lateral ventricles, mainly of the fourth ventricle; and severe disorganization between the gray matter and white matter (Fig. 2a). No regional and anatomical alterations in cerebral structures were found in all healthy control children at time of the blood sample collection (Fig. 2b).

### Electroencephalogram

**Fig. 3:**
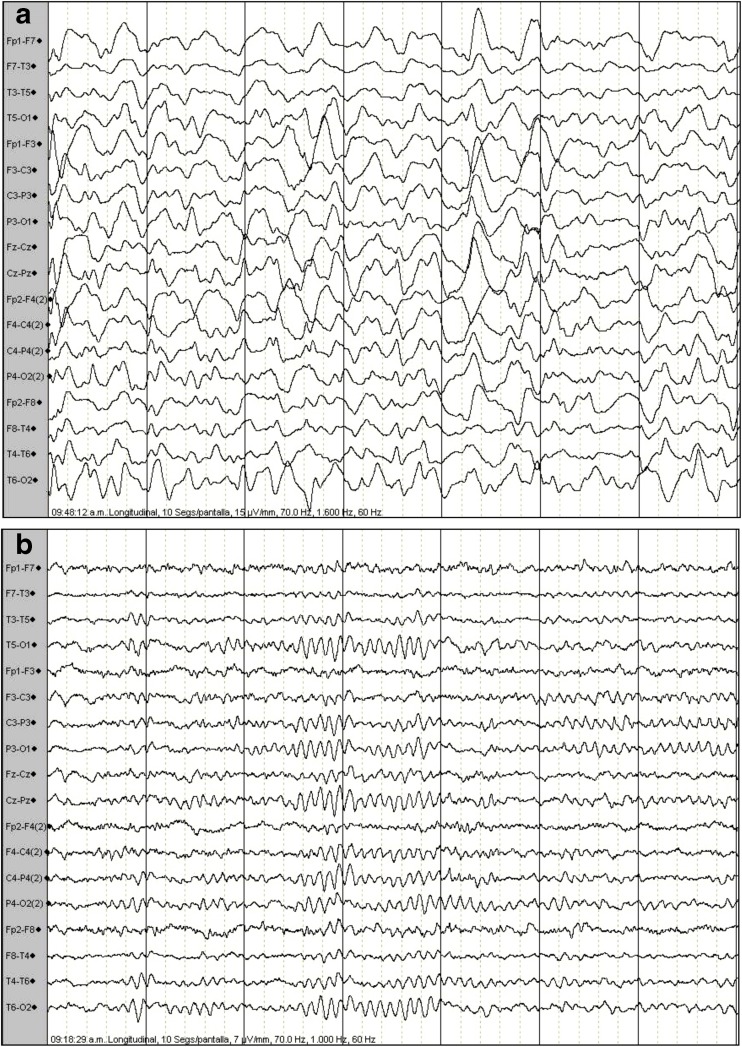
Representative electroencephalograms. Cerebral palsy patient (**a**) and a healthy control child (**b**)

The EEG neonatal records of CP patients showed multiple seizures (25%), low amplitude seizures, rare spikes (18.7%), low voltage rare spikes (16.7%), bilateral epileptiform spikes (16.7%), sharp-waves (10.4%), rare spikes, mild suppression (8.3%), and severe suppression (4.2%), while current EEG records showed slow and high amplitude waves and epileptiform activity, mainly including acute waves, spike-waves, and a complex of polyspike-waves in the frontal and temporal records in more than 81% of all patients with CP (Fig. 3a). In contrast, the healthy control children did not show electrophysiological alterations (Fig. 3b).

### Allele Distribution of the rs3833912 Polymorphism of NOS2A

**Fig. 4:**
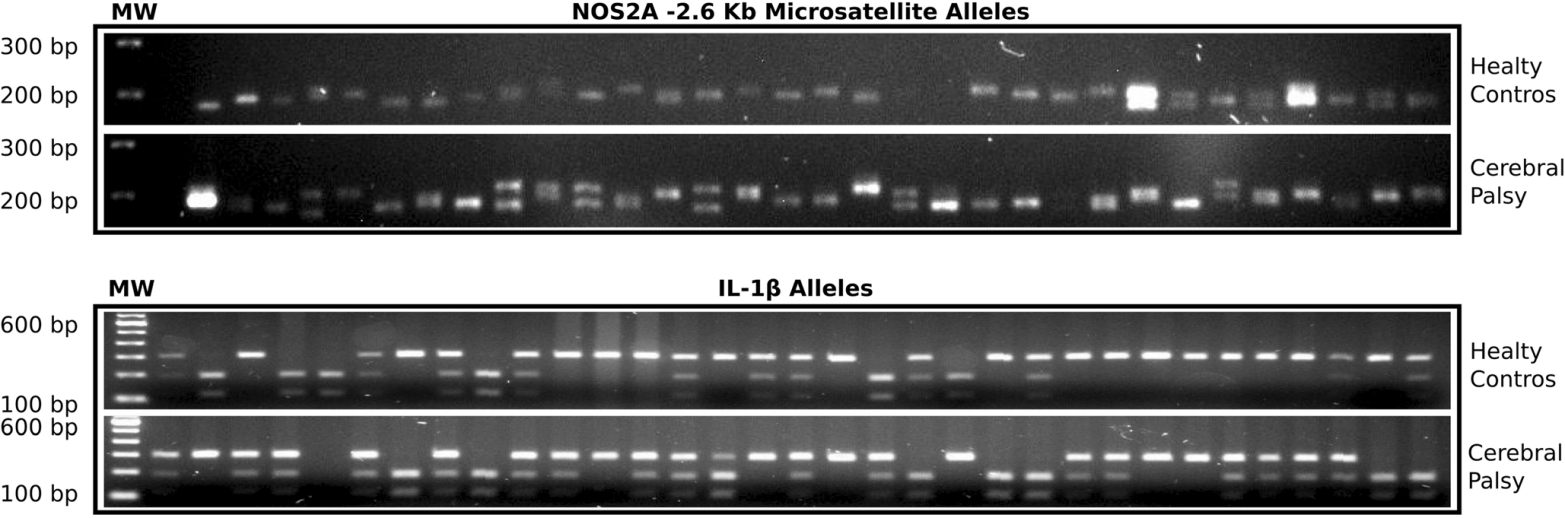
Electrophoretic pattern of the NOS2 gene promoter microsatellite CCTTT and IL-1β gene promoter SNP -511 polymorphism. **a** PCR product for the genomic region surrounding the (CCTTT)_n_ polymorphic microsatellite. **b** RFLP results using the AvaI restriction enzyme. The homozygous C/C genotype shows a single band of 273 bp, the homozygous T/T genotype shows two bands of 100 and 173 bp, and the heterozygous C/T genotype shows three bands of 100, 173, and 273 bp. MW = molecular weight markers

After review of the HapMap database, we did not find an overall incidence of the CCTTT polymorphic microsatellite of the NOS2 gene promoter in the general Mexican population. We detected 13 different alleles in microsatellite CCTTT (rs3833912), which is localized at − 2.5 kb of the NOS2A gene promoter (Fig. 4). The expansion ranged from 5 to 17 repeats (Fig. 4) following a Gaussian distribution with the following values: the frequency reached 0.3% at 5 repeats and 0.1% at 17 repeats, with a peak of 15% at 14 repeats (Table 3). For statistical analysis, alleles were grouped into three classes: short genotypes (5 to 12 repeats), physiological genotypes (13 and 14 repeats), and long genotypes (15 to 17 repeats; Table 4). The analysis of the 13 repeat-long and 14 repeat-long genotypes (physiological alleles) was carried out independently (Table 4). Short-form genotypes (< 12 repeats) were present in 60.4% of CP patients (*n* = 29) and in 68.4% of healthy controls (*n* = 39; Table 4). The physiological 13 repeat-long genotype was present in 6.3% of CP patients (*n* = 3) and in 8.8% of the healthy control group (*n* = 5), whereas the 14 repeat-long genotype was present in 27% of CP patients (*n* = 13) and in 12.3% of healthy controls (*n* = 7). Long-form genotypes (> 15 repeats) were present in 6.3% of CP patients (*n* = 3) and in 10.5% of healthy controls (*n* = 6; Table 4). Statistical analysis revealed that short-form genotypes and 13 repeats of the CCTTT microsatellite did not affect the predisposition for developing CP (OR = 0.7045, 95% CI = 0.3152–1.5742, *P* = 0.2575); in the same manner, the physiological 14-repeat CCTTT genotype was not significantly associated with the development of CP in patients with a history of perinatal hypoxia-ischemia (OR = 2.6531, 95% CI = 0.9912–7.3232, *P* = 0.0469).

**Table 3:** Allele distribution of rs3833912 polymorphisms in the NOS2A gene promoter

CCTTT repeated number	Cerebral palsy					Healthy controls				
	Allele frequency		Total	Genotype frequency		Allele frequency		Total	Genotype frequency	
	Allele 1	Allele 2		Homozygotes	Heterozygotes	Allele 1	Allele 2		Homozygotes	Heterozygotes
5	1	1	2	0.0033	0.0100	1	1	2	0.0067	0.0033
6	3	1	4	0.0067	0.0133	3	1	4	0.0167	0.0167
7	1	1	2	–	0.0067	1	1	2	0.0031	0.0200
8	2	2	4	–	0.0400	2	2	4	0.0167	0.0367
9	4	3	7	0.0067	0.0733	3	3	6	0.0367	0.0333
10	6	7	13	0.0067	0.1000	6	7	13	0.0367	0.0767
11	3	5	8	0.0067	0.0600	4	8	12	0.0533	0.0367
12	10	8	18	0.0733	0.1067	7	10	17	0.0733	0.0733
13	7	7	14	0.0433	0.0800	8	3	11	0.0567	0.0933
14	11	15	26	0.1567	0.0733	7	6	13	0.0967	0.0567
15	6	4	10	0.0267	0.0567	2	3	5	0.0567	0.0033
16	2	2	4	0.0033	0.0333	3	2	5	0.0200	0.0567
17	1	1	2	0.0033	0.0100	1	1	2	0.0167	0.0033
**Total**	**57**	**57**	**114**	**0.3367**	**0.6633**	**48**	**48**	**96**	**0.4900**	**0.5100**

**Table 4:** Genotype distribution of rs3833912 polymorphisms in the NOS2A gene promoter

Genotype	(CCTTT)n	Groups		*P* value	RR	95% CI	OR	95% CI
		Cerebral palsy	Healthy controls					
Short	*n ≤ 12*	29 (60.4%)	39 (68.4%)	0.2575	0.88	0.66–1.17	0.70	0.31–1.57
Physiological	*n = 13*	3 (6.3%)	5 (8.8%)	0.4579	0.71	0.17–2.82	0.69	0.15–3.06
	*n = 14*	13 (27.0%)	7 (12.3%)	0.0469	2.20	0.95–5.08	2.65	0.96–7.32
Long	*n ≥ 15*	3 (6.3%)	6 (10.5%)	0.3374	0.59	0.15–2.24	0.56	0.13–2.39

### Allele and Genotype Distribution of the rs16944 Polymorphism of IL-1β

Allelic discrimination analysis showed a frequency of 62.5% for allele T and of 37.5% for allele C in patients with CP (Fig. 4 and Table 5). The healthy control group showed similar rates of 50.8% for allele C and 49.2% for allele T (Fig. 4 and Table 5). The allelic frequency of the -511 polymorphism of the IL-1β gene promoter agreed with those in the HapMap database. Genotype analysis for -511C-T (rs16944) showed a low frequency of the homozygous CC genotype in CP patients (16.7%) compared with healthy controls (26.3%; *P* = 0.1702, OR = 0.5600, 95% CI 0.2142–1.4642 and RR = 0.6333, 95% CI 0.2941–1.3643; Table 6). The frequencies of heterozygous CT and TC were also lower in CP patients (39.6%) than in healthy controls (50.9%; *P* = 0.1684, OR = 0.6326, 95% CI 0.2937–1.3764 and RR = 0.7780, 95% CI 0.5047–1.1992; Table 6). In contrast, the homozygous TT genotype was higher in CP patients (43.8%) than in the healthy controls (22.8%; *P* = 0.0189, OR = 2.6325, 95% CI 1.1348–6.1066 and RR = 1.9183, 95% CI 1.0791–3.4102; Table 6).

**Table 5:** Allele frequency of rs16944 polymorphisms of the IL-1β gene promoter

IL-1β gene	Allele	Number of patients per group		Statistics	
		Cerebral palsy (*n* = 48)	Healthy controls (*n* = 57)	*P*	OR (95% CI)
**rs16944**	**C**	**36 (0.375)**	**58 (0.508)**	0.0114^a^	0.55 (0.44–0.70)
	**T**	**60 (0.625)**	**56 (0.492)**		

^a^Statistically significant difference

**Table 6:** Genotype distribution of rs16944 polymorphisms of the IL-1β gene promoter

Genotype	Groups		*P* value	RR	95% CI	OR	95% CI	HWE
	Cerebral palsy	Healthy controls						
CC	8 (16.7%)	15 (26.3%)	0.1702	0.63	0.29–1.36	0.56	0.21–1.46	0.518
**TT**	**21 (43.8%)**	**13 (22.8%)**	**0.0189** ^**a**^	**1.91**	**1.07–3.41**	**2.63**	**1.13–6.10**	**0.531**
CT	19 (39.6%)	29 (50.9%)	0.1684	0.77	0.50–1.19	0.63	0.29–1.37	0.594

*HWE* Hardy-Weinberg equilibrium

^a^Statistically significant difference

### Association of rs3833912 and rs16944 with Cerebral Palsy Development

The haplotype (CCTTT)_≤12_/CC showed a frequency of 16.7% in the group of patients with CP vs 15.8% in the healthy control group (Table 6). The haplotype (CCTTT)_≤12_/TT was present in ≈ 14% in both groups, and the haplotype (CCTTT)_≤12_/CT was dominant in the healthy control group (42.1%) with respect to CP patients (13%). However, no statistically significant difference in the three haplotypes was found (Table 7). The combination (CCTTT)_13_/CC, (CCTTT)_13_/TT, and (CCTTT)_13_/CT showed a low frequency, from 2.1 to 4.2%, representing one or two patients in each group studied, and statistical analysis of these groups revealed no significant difference between CP patients and healthy controls (Table 6). The haplotypes (CCTTT)_14_/CC and (CCTTT)_14_/CT showed a similar frequency of ≈ 3.5 and ≈ 7%, respectively, in both groups (Table 7). However, haplotype (CCTTT)_14_/TT was present in 14.6% of patients with CP, whereas in the healthy control group, it was lower than 1.8% with respect to all participants, showing a statistically significant difference (*P* = 0.01, RR = 8.3, CI = 1.05–65.20 and OR = 9.5, CI = 1.13–80.75 and Hardy-Weinberg equilibrium = 0.591). Finally, the haplotypes (CCTTT)_≥15_/CC, (CCTTT)_≥15_/TT, and (CCTTT)_≥15_/CT were present in low frequency, from 1.8 to 3.5%, accounting for one or two patients in the groups studied (Table 7). Statistical analysis revealed no significant difference between the two groups studied (Table 7). The correlation analysis between the Sarnat score and different polymorphisms studied did not show significant differences because patients with Sarnat III were only 27% of all patients studied, and not all of the microsatellite polymorphisms occurred in this classification (data not shown).

**Table 7:** Cerebral palsy risk for the combination of the − 2.5 kb (CCTTT)n microsatellite of the NOS2A gene promoter (rs3833912) and the IL-1β -C511 → T SNP (rs16944)

Polymorphism		Groups		Haplotype association					
rs3833912	rs16944	Cerebral palsy	Healthy controls	*P* value	RR	95% CI	OR	95% CI	HWE
*(CCTTT)≤12*	CC	8 (16.7%)	9 (15.8%)	0.5551	1.05	0.44–2.52	1.06	0.37–3.02	0.361
	TT	7 (14.6%)	8 (14.0%)	0.5766	1.03	0.40–2.65	1.04	0.34–3.12	0.619
	CT	13 (27.1%)	24 (42.1%)	0.0802	0.64	0.36–1.12	0.51	0.22–1.16	0.814
*(CCTTT)13*	CC	1 (2.1%)	2 (3.5%)	0.5647	0.59	0.05–6.34	0.58	0.05–6.65	0.432
	TT	1 (2.1%)	1(1.8%)	0.7076	1.18	0.07–18.45	1.19	0.07–19.57	0.594
	CT	2 (4.2%)	2 (3.5%)	0.6235	1.18	0.17–8.11	1.19	0.16–8.82	0.736
***(CCTTT)14***	CC	2 (4.2%)	2 (3.5%)	0.6235	1.18	0.17–8.11	1.19	0.16–8.82	0.501
	**TT**	**7 (14.6%)**	**1 (1.8%)**	**0.0164** ^**a**^	**8.31**	**1.05–65.20**	**9.56**	**1.13–80.75**	**0.591**
	CT	4 (8.3%)	4 (7.0%)	0.5420	1.18	0.31–4.49	1.20	0.28–5.09	0.676
*(CCTTT)≥15*	CC	1 (2.1%)	1(1.8%)	0.7076	1.18	0.07–18.48	1.19	0.07–19.57	0.255
	TT	1 (2.1%)	1(1.8%)	0.7076	1.18	0.07–18.48	1.19	0.07–19.57	0.451
	CT	1 (2.1%)	2 (3.5%)	0.5647	0.59	0.05–6.34	0.58	0.05–6.65	0.672

*HWE* Hardy-Weinberg equilibrium

^a^Statistically significant difference

## Discussion

Our results showed for the first time that haplotype (CCTTT)_14_/TT in the promoters of the NOS2 and IL-1β genes in Mexican children who had suffered from perinatal HIE had a major association with the risk for developing CP. Previous studies have suggested that the development of CP may be more frequent in patients who presented with a sustained low Apgar score, which indirectly reflected a widespread deprivation of oxygen to the brain [22]. Accordingly, in our study, patients with CP had sustained low Apgar scores during the first 10 min after delivery. However, the Apgar score alone is not a sufficient criterion to diagnose cerebral hypoxia and predict neurological sequels because only 11% of children with an Apgar score of less than 3 at birth were diagnosed with CP [23]. In our study, the Apgar score was complemented in 73% of our CP patients with cord blood gas determinations. In all samples measured, cord blood gas analysis showed the presence of metabolic acidosis, suggesting that CP was a sequel of HIE.

Our EEG and CAT scan results supported the neurological diagnosis of CP. Quadriparesis was the most common type of paralysis that we assessed in the Mexican children studied. These findings showed damage to the descending motor pathways (the upper-motor neuron syndrome) that might have occurred because of HIE, as suggested by the sustained low Apgar scores and cord blood gas results. In addition, we determined that 54% of the CP patients presented with quadriparesis. This finding agreed with results obtained in a Canadian population [24] and disagreed with other prospective series in which problems in learning and memory were the most representative of hypoxia/ischemia [25]. The disagreement can be explained by the difference in the design of study. In our study, the patient group was selected considering an established CP, medical records of HIE, and low Apgar scores. This is why the presence of spastic quadriparesis was the predominant subtype.

High levels of NO from NOS2 activity have been implicated in the deleterious effects on the central nervous system [3, 26]. A previous study of diabetic retinopathy in vitro has demonstrated an increased transcriptional activity of NOS2A when the (CCTTT)_14_ allele is present, in comparison with the alleles (CCTTT)_9_, (CCTTT)_12_, and (CCTTT)_15_ [13]. Our data agree with those findings and provide support to our proposal that an expansion to 14 repeats of the CCTTT microsatellite increases the risk of developing CP in newborns with a history of HIE.

This observed increase in the susceptibility to developing CP in the carrying patients of the genotype (CCTTT) 14 is based on a weak correlation with a marginal statistics value, so the change in the size of the sample is a critical factor to validate this polymorphism such an individual marker.

However, in some other pathologies such as diabetic retinopathy, the expansion of the microsatellite CCTTT to 14 repeats has been correlated as a protective factor, because the retina of diabetic patient requires an overproduction of nitric oxides that delay or prevent microvascular complications of diabetes [13].

IL-1β has also been implicated in the brain induced under hypoxia-ischemia conditions [27], although the molecular mechanism remains unknown. Previous studies have shown that the rs16944 polymorphism controls IL-1β transcription, especially the genotypes TT and CT, which have been involved in IL-1β overproduction under inflammatory conditions [28]. The promoter SNP locus rs16944 (-511 C > T) is located in a regulatory motif. This mutation, facilitating interactions with transcriptional factors, increases the activity of the IL-1β gene promoter [29]. This mechanism might account for the association of the TT genotype at the -511 position of the IL-1β gene promoter with increased risk for developing CP after HIE observed in Mexican children.

In this work, we found no statistical association among the short and long form of the rs3833912 NOS2A microsatellite (< 12, 13, and > 15 repeats) and different genotypes of the rs16944 IL-1β polymorphism with CP. The presence of the shorter 12-repeat microsatellite allele might be responsible for normal or low NO production, possibly by the poor response of the NOS2A gene promoter to IL-1β after an event of perinatal asphyxia. This possibility might explain the decreased risk of developing CP in individuals who have short-form CCTTT microsatellite alleles. The individual assessment of (CCTTT)_14_ genotype showed an individual low increased risk of developing CP in Mexican children after HIE. Interestingly, the haplotype CCTTT_14_/TT analysis showed a potentiation in the global risk of cerebral palsy development following HIE. Supporting this finding recently has been reported that the combined inhibition of nNOS and iNOS started as soon as possible after birth and in a repeated dosing regimen seems to have the best potential based on the combined outcome parameters, translation to clinical practice, and methodological quality [29]. By this reason, we propose that the CCTTT_14_/TT haplotype could be a useful marker to identify patients who will require pharmacological treatment with iNOS inhibitors and a strong early stimulation program to induce the synaptic plasticity process in the cerebral cortex in order to decrease the severity of neurological sequels following the HIE and try to avoid the development of cerebral palsy.

**Fig. 5:**
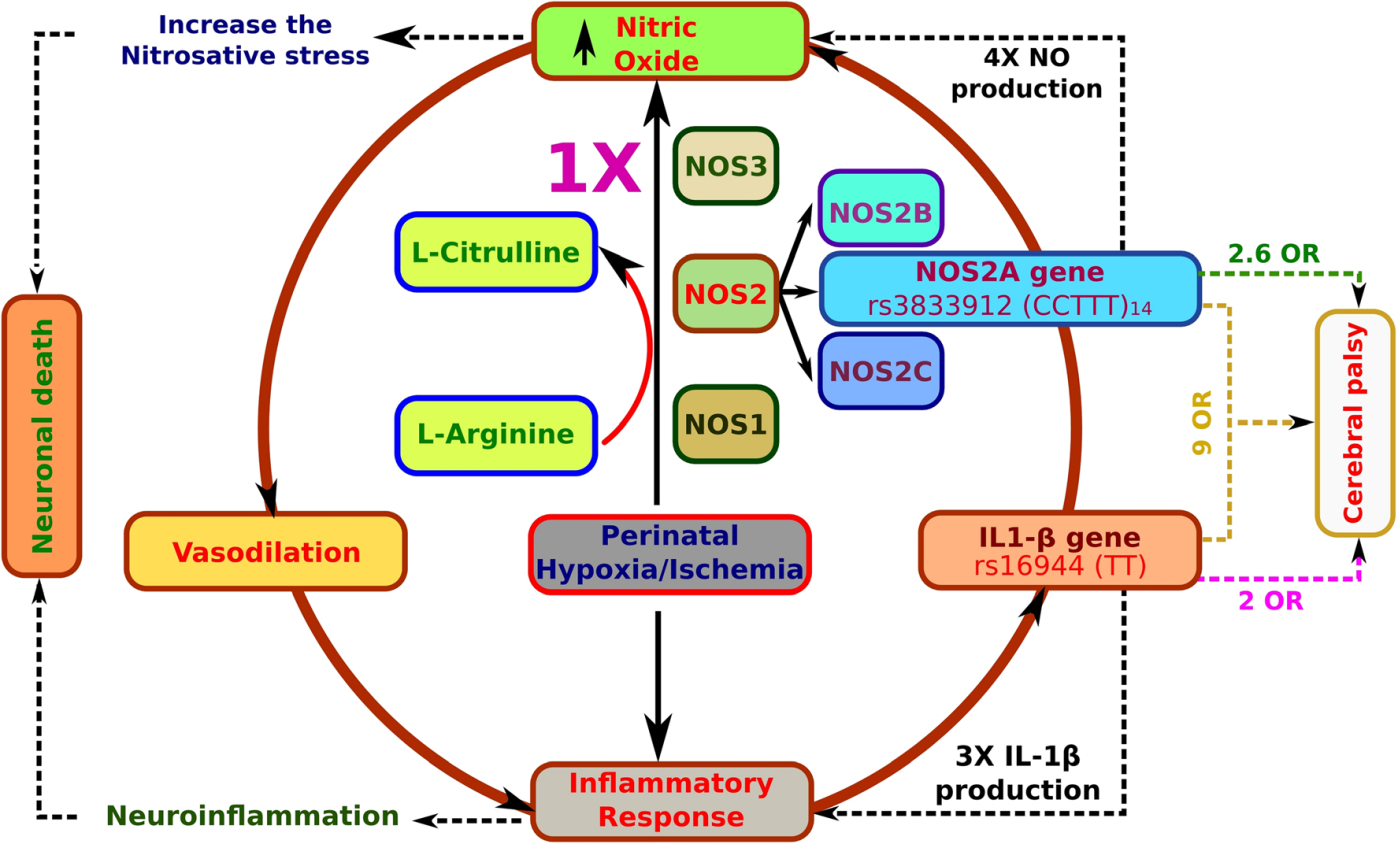
Diagram suggesting the possible association between rs3833912 and rs16944 polymorphisms and the risk for cerebral palsy. Following a perinatal asphyxia event, the normal brain responds by increasing NO production through constitutive NOS isoforms (nNOS or NOS1 and eNOS or NOS3) to enhance brain perfusion. At the same time, neuroinflammation increases IL-1β production. In contrast, patients who had the haplotype CCTTT14/TT responded with an uncontrolled production of NO through an inducible isoform of NOS (iNOS or NOS2) and a further increase of IL-1β production, which might aggravate the damage in the immature cerebral cortex, thus contributing to the development of CP as a sequel of the perinatal asphyxia. OR = odds ratio

Based on our data, we propose a hypothetical mechanism to explain the possible role of the haplotype (CCTTT)14/TT in the -2600 and -511 positions in the promoters of iNOSA and IL-1b genes in the development of CP following an event of neonatal HIE (Fig. 5).

## Conclusions

The haplotype (CCTTT)14/TT, formed by the expansion of the − 2.5 kb (CCTTT)n microsatellite in the NOS2A gene promoter and the -511 C ➝ T SNP of the IL-1β gene promoter, has the potential to be used as a genomic marker to identify high risk patients to developing cerebral palsy as a sequel to neonatal hypoxic-ischemic encephalopathy. However, this should be taken with high caution due to the size of the studied sample. It is convenient to make a replicate in a prospective study, where the neonates must be recruited, studied with blood analysis, EEG, CAT scan, or magnetic resonance imaging (MRI), and genotyped during the HIE and healthy controls and following them during 5 years in order to validate the power of CCTTT14/TT haplotype to predict cerebral palsy development.

## Abbreviations

CAT: Computerized axial tomography
CI: Confidence interval
CP: Cerebral palsy
CRIT: Comprehensive Rehabilitation Center Telethon
DNA: Deoxyribonucleic acid
EEG: Electroencephalogram
HIE: Hypoxic ischemic encephalopathy
HWE: Hardy-Weinberg equilibrium
IL-1β: Interleukin-1 beta
NICU: Neonatal intensive care unit
NO: Nitric oxide
NOS1 or nNOS: Nitric oxide synthase type 1 or neuronal
NOS2A or iNOS: Nitric oxide synthase type 2A or inducible
NOS3 or eNOS: Nitric oxide synthase type 3 or endothelial
SNP: Single nucleotide polymorphism
OR: Odds ratio
PCR: Polymerase chain reaction
RR: Relative risk
RFLP: Restriction fragment length polymorphism
